# A Presumed Metastatic Lung Lesion Unveiling a Second Primary: Renal Cell Carcinoma With Coinciding Invasive Mucinous Adenocarcinoma

**DOI:** 10.7759/cureus.107594

**Published:** 2026-04-23

**Authors:** Kevin T Dao, Sunjum Singh, Parmveer Kaloty, Danial Bandak, Mia Yasonova, Harmanjeet Dhillon, Chandpreet Singh, Wilbur Montana

**Affiliations:** 1 Internal Medicine, UCLA (University of California Los Angeles) Kern Medical, Bakersfield, USA; 2 Hematology and Medical Oncology, UCLA (University of California Los Angeles) Kern Medical, Bakersfield, USA

**Keywords:** invasive mucinous adenocarcinoma of the lung, multiple primary malignancies, renal cell carcinoma, separate malignancies, surgical intervention, systemic medical therapy

## Abstract

Multiple primary cancer is a self-explanatory term, albeit very uncommon. In most instances, when patients have a malignancy, the possibility of metastasis should be considered. Renal cell carcinoma (RCC) is one such cancer. However, as unlikely as it is, there is always a chance that another separate, unrelated malignancy could be present. Here, we would like to discuss a unique case of a 66-year-old female who not only developed primary RCC but also primary invasive mucinous adenocarcinoma of the lung. This revelation prompted a change in management, resulting in the medical team treating the two malignancies separately via surgical intent as opposed to treating metastatic RCC, which is more geared toward systemic medical therapy.

## Introduction

Renal cell carcinoma (RCC) is a common kidney malignancy, predominantly seen in male patients, with various subtypes and a mortality rate of approximately 30-40% [1]. Although more patients are being diagnosed in early stages of the disease, 20-30% of these cases are already metastatic at presentation [1]. Fortunately, with the advancement of imaging techniques, there has been an increase in detection and incidental findings of RCC [2]. As of now, the first stages of RCC can be managed with partial/radical nephrectomy, whereas the later stages require either a combination of surgical resection and neoadjuvant/adjuvant therapy, with stage 4 requiring systemic therapy [1-3].

In contrast, lung cancer is one of the leading causes of malignancy-related mortality worldwide and is primarily divided into either small cell lung cancer or non-small cell cancer [4,5]. Pulmonary adenocarcinoma is the most common non-small cell lung cancer and can be further divided into various subtypes [5]. One such subtype is invasive mucinous adenocarcinoma (IMA) of the lung, which accounts for approximately 2-10% of all pulmonary adenocarcinomas [5,6]. This type of pulmonary malignancy is characterized by invasive growth of columnar or goblet cells with abundant intracytoplasmic mucin, usually in the peripheral regions and particularly in the lower lobes of the lungs [7]. Furthermore, IMA of the lung has a distinct lepidic pattern on histology, which distinguishes it from other lung adenocarcinomas [7,8]. Unfortunately, the prognosis of patients who are diagnosed with IMA of the lung is unclear due to the low incidence [9].

When looking at the probability of having both RCC and IMA of the lung, even fewer cases are to be found. One study showed that the incidence of having multiple primary malignancies of any kind in a population of cancer patients varied between 2.4% and 8% and can even increase to 17% over a course of 20 years [10], whereas another study claimed this percentage is increasing with advancements in treatment and earlier oncological detection, contributing to longer survivorship [11]. However, since malignancies can metastasize, it can be difficult to make the diagnosis of multiple primary cancers. For example, in RCC, the risk of metastases varies from 3.6% to 45.1%, and RCC itself has been known to have distant metastasis to the bone, brain, and lungs [1,12]. Thus, in patients with RCC presenting with a lung nodule, it is more likely to assume the nodule is from metastatic disease rather than an additional primary tumor. Although a fair conclusion, the common pitfall and assumption can lead to improper medical treatment.

In this study, we present a unique case of a patient who had RCC and was found to have invasive mucinous carcinoma of the lung upon biopsy of a nodule seen incidentally on CT scan, originally thought to be a metastasis. Due to this, the originally planned treatment courses for metastatic RCC had to be changed to manage both separate primary malignancies. Here we would like to discuss how crucial it is to verify the diagnosis via confirmatory measures, as well as to discuss the need for a biopsy to help further guide management.

## Case presentation

**Figure 1:**
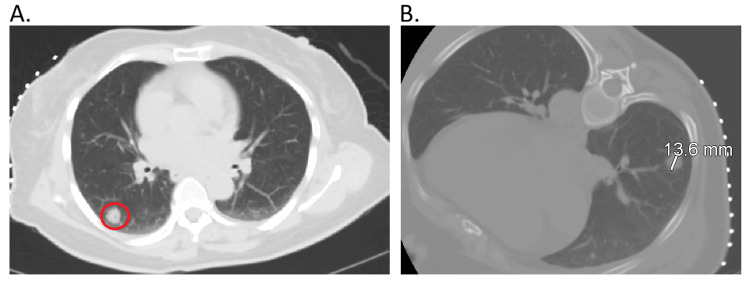
Computed tomography of the chest, abdomen, and pelvis with contrast A. The red circle indicates a subsolid nodule in the right lower pulmonary lobe. B. The same nodule is noted to be 13.6 mm in diameter.

The patient is a 66-year-old woman who presented initially as a consult to the hematology and oncology clinic for evaluation and treatment of a prediagnosed RCC from another facility with concerns of possible pulmonary metastasis. The patient stated that she had plans for a tentative partial nephrectomy and had a repeated computed tomography (CT) of the chest, abdomen, and pelvis (Figure 1A, 1B). The primary care physician (PCP) then referred the patient to hematology and oncology after noting the pulmonary nodule at the follow-up PCP clinic.

**Figure 2:**
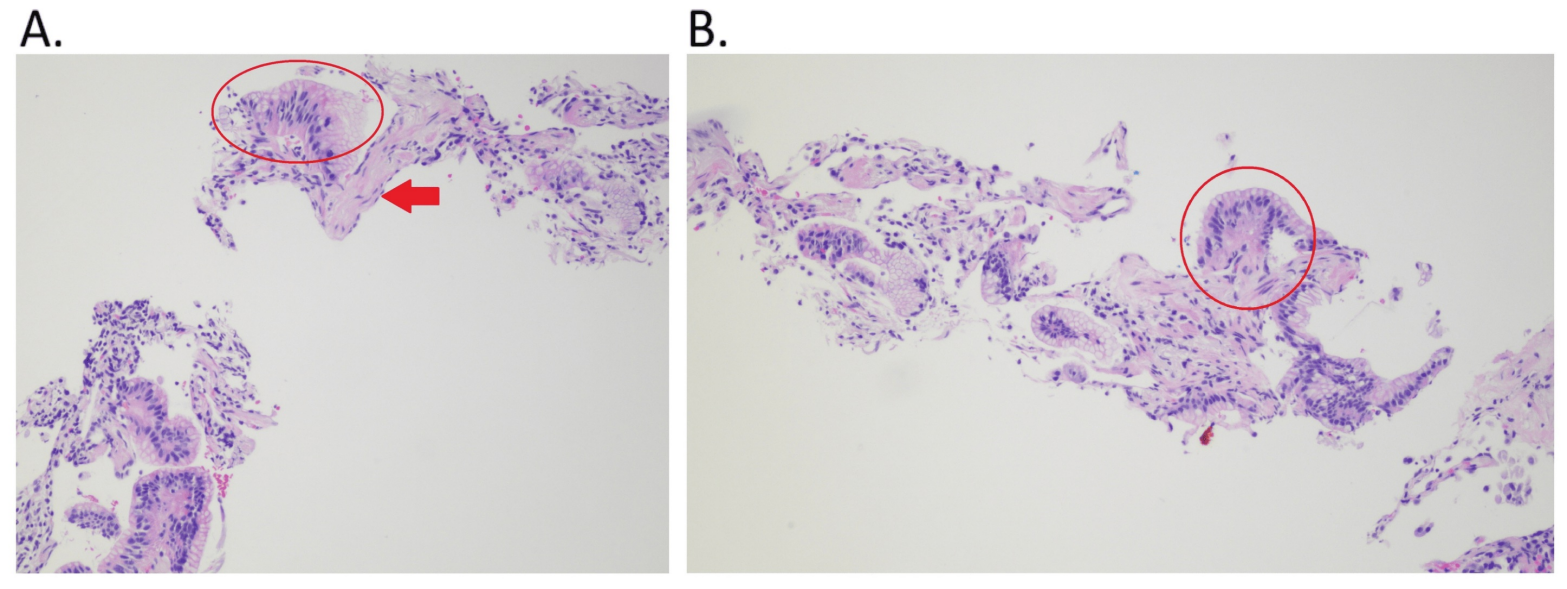
Needle core biopsy of the right lower lobe mass A. The red circle indicates a lepidic pattern of abnormal cells with the red arrow indicating normal pneumocytes. A few areas show atypical pneumocytes that are positive for CK7. TTF1 is focally positive and is negative for PAX8, CK20, and CDX2. B. This is a different picture of the same path slide with the red circle showing the same lepidic pattern of abnormal cells.

On evaluation at the oncology clinic, she stated that she had been having focal left non-radiating flank pain and described it as a persistent dull ache for over one month that would come and go. She endorsed that this pain began to resolve spontaneously and is now only present intermittently. The patient otherwise denies any frank hematuria, dysuria, incontinence, urgency, chest pain, hemoptysis, or any other symptoms at this current time. The vitals, physical exam, and basic blood work were unremarkable, and the patient was scheduled for a lung nodule biopsy. After the lung nodule biopsy, the pathology reports were reviewed at a follow-up oncology appointment, which showed atypical scant pneumocytes with no definitive diagnosis given. However, immunohistochemistry was positive for CK7 and TTF1 and negative for PAX8, CK20, and CDX2, which favors a lung primary versus a biopsy showing no definitive diagnosis (Figure 2A, 2B). The plan from oncology was that the patient was scheduled for a tentative partial nephrectomy at her higher level of care facility, in addition to an appointment with pulmonology and a repeat lung biopsy.

The patient then went to her pulmonology appointment first, and a multidisciplinary discussion was held with both pulmonology and hematology/oncology. It was noted that the lung primarily showed solid ground-glass nodularity congruent with clinical adenocarcinoma in situ (AIS), with other differentials being atypical adenomatous hyperplasia and the inability to exclude unsampled invasion. Both specialties agreed that a repeat CT-guided biopsy was needed to accurately determine if the patient had metastatic RCC or two separate primary malignancies, which would guide medical management. However, if the biopsy results were inconclusive once more, then the plan would be to send the patient for definitive resection for an accurate diagnosis. Shortly after, a repeated lung needle core biopsy was done at another facility. In the interim, the patient was still scheduled for a tentative partial nephrectomy and was pending a follow-up appointment with hematology/oncology to discuss the pathology results of the lung needle core biopsy.

**Figure 3:**
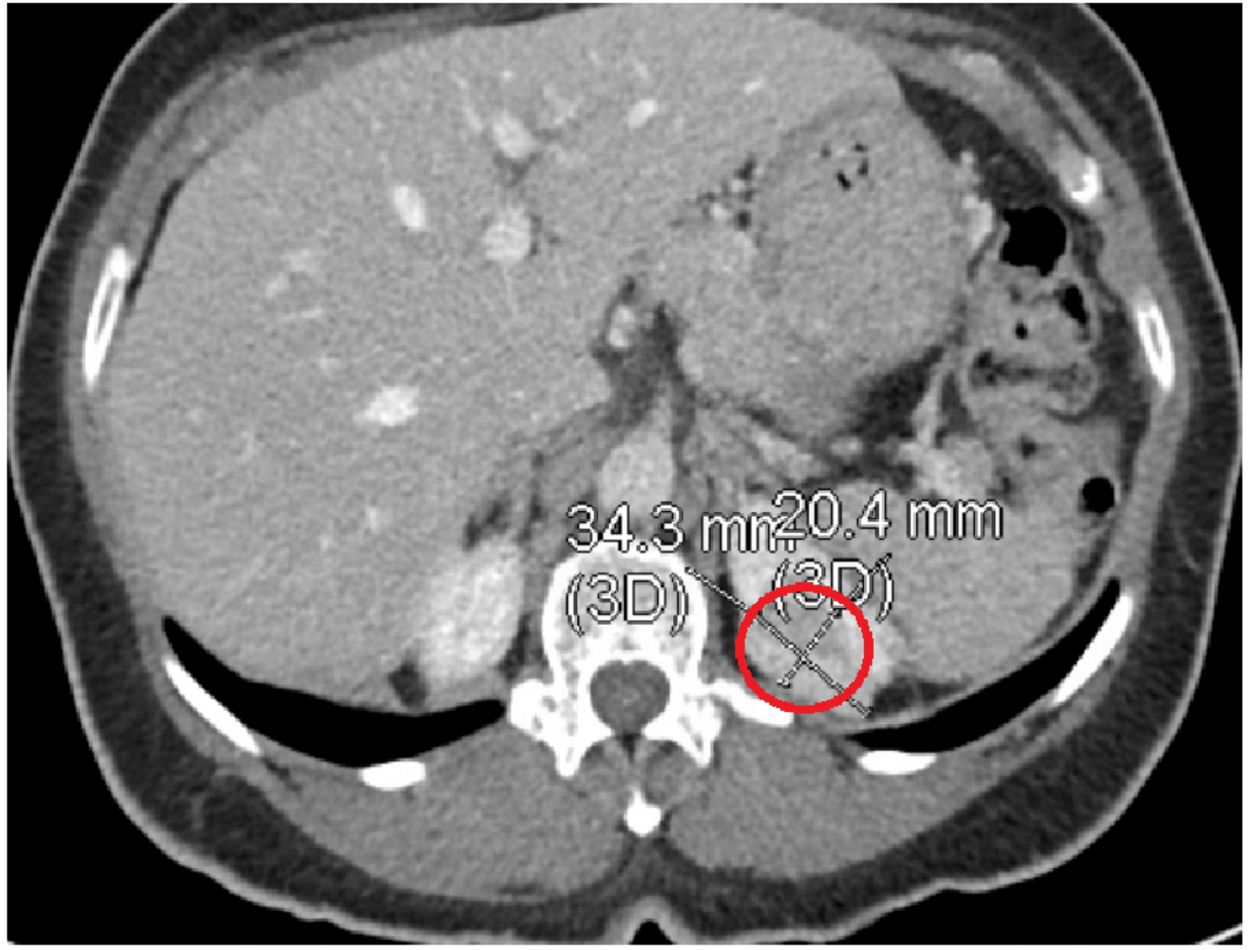
Computed tomography of the abdomen and pelvis with contrast The figure shows a poorly defined enhancing solid left superior renal upper pole with a posterior cortical mass about 34.3 mm × 20.4 mm in size, further highlighted by the red circle. This has extended beyond the renal capsule.

**Figure 4:**
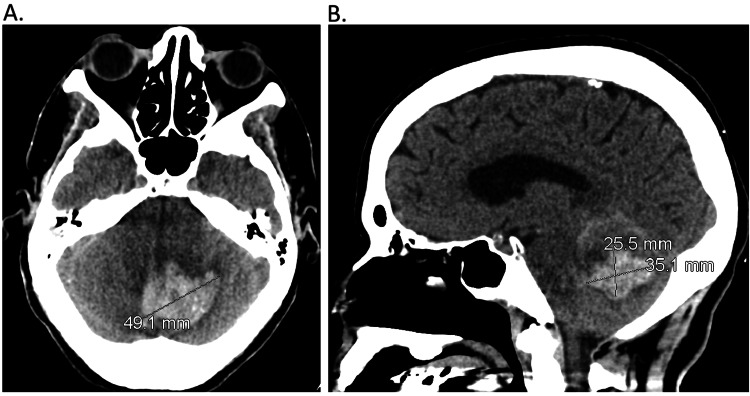
Computed tomography of the brain and head without contrast A. Transverse view showing a 49.1 mm hematoma in the cerebellum. B. Sagittal view showing a 25.5 × 35.1 mm hematoma in the cerebellum. Hemorrhage is noted and extends into the fourth ventricle with dilatation of the temporal horns suggesting probable early hydrocephalus.

Unfortunately, the patient's husband stated that she had a severe headache along with nausea and vomiting. The vitals showed systolic blood pressure in the 170s to 180s with diastolic in the 80s to 90s. The patient had a CT abdomen and pelvis with contrast that showed a poorly defined, enhancing solid left renal upper pole posterior cortical mass about 34.3 mm × 20.4 mm in size (Figure 3). A brain and head CT without contrast was ordered due to headache and vomiting, which showed cerebellar hemorrhage (Figure 4A, 4B). She was then taken to the operating room with neurosurgery for posterior fossa craniectomy and evacuation of the clot vs. brain mass, and the ICU was consulted for post-op management. The pathology results from the blood clot showed no evidence of malignancy, but rather a fresh blood thrombus. In the interim, records were requested from the other facility where the right lung needle core biopsy was done, which showed IMA consistent with primary IMA of the lung. As such, the patient was diagnosed with RCC stage III T3a Nx M0 and IMA stage IA2 T1b Nx M0 due to unknown nodal involvement but no metastasis. This underlying result of two primary malignancies with no other lesions or masses present shifted the treatment to a more surgical intention. Unfortunately, she was unable to be extubated and remained intubated with waxing and waning mentation. A discussion was held with the patient’s husband, and after a percutaneous tracheostomy as well as a percutaneous endoscopic gastrostomy (PEG) tube placement, the patient was discharged to a long-term acute care facility.

## Discussion

Like most malignancies, management depends on the staging of the disease. RCC typically requires surgical intervention; however, when the disease progresses to stage 4, systemic medication therapy is needed [2]. In cases where systemic therapy is necessary, the type of medication can be VEGF inhibitors, tyrosine kinase inhibitors, immunotherapy, etc. [13]. In this case, the patient was noted to have a pulmonary nodule that showed concern for metastatic RCC, with prior studies noting management based on the size of the nodules [14]. Although metastatic RCC was the most likely diagnosis in our patient, the possibility of two unrelated primary malignancies remained; therefore, a biopsy was performed. The patient's needle core biopsy was noted to be positive for CK7, with TTF1 focally positive and negative for PAX8, CK20, and CDX2, which highly favors a lung primary in nature [15,16]. These findings led to a diagnosis of primary IMA of the lung. This was unexpected, given that metastatic RCC was statistically more probable than the presence of two separate primary tumors [1,10-12]. Despite this, some cases have been documented with varying management as to how to approach multiple unrelated malignancies. One study noted successful first-line management of both lung adenocarcinoma and renal clear cell carcinoma with albumin-bound paclitaxel and cisplatin in combination with sintilimab [17], whereas another case report had treated the patient with primary clear cell RCC and metastatic adenocarcinoma with furmonertinib and radiotherapy [18]. In rare instances, patients may develop three distinct malignancies; one report describes metachronous primary lung adenocarcinoma and renal mucinous tubular and spindle cell carcinoma treated with surgery and chemotherapy [19]. These unique cases of multiple primary cancers further elucidate the importance of knowing whether or not the patient has metastatic disease or multiple primaries. Furthermore, knowing the exact primary malignancy is also key since various studies have shown cancers to each have their own treatment course. In our case, assuming the patient had metastatic disease would have been reasonable based on the likelihood of metastatic disease versus multiple primaries; however, it would have promoted an incorrect treatment. This would have resulted in the patient being treated for metastatic RCC while still having an underlying lung primary that could progress. In regard to IMA of the lung, prior studies show that surgical intervention for resectable IMA has a higher overall survival compared to patients with noninvasive mucinous adenocarcinomas [9]. Thus, for our patient, the medical management was more geared to the surgical intention of both the kidney and pulmonary nodule as opposed to systemic medical therapy [4,13]. Unfortunately, the patient had developed complications from her hypertension, leading to a hemorrhagic stroke; therefore, the patient wasn’t able to have the treatment regarding her malignancies. Despite this, our case itself shows a unique presentation of a patient having two separate primary malignancies and depicts the importance of ensuring that the patient has metastatic RCC as opposed to two separate primary malignancies, since the diagnosis changes management.

## Conclusions

Management of metastatic RCC involves systemic medication therapy (tyrosine kinase inhibitors, immunotherapy, etc.), which differs from non-metastatic RCC and non-metastatic IMA of the lung, which would include more surgical intervention. Although it would have been reasonable to assume that the patient had metastatic RCC, this case shows that the biopsy results are crucial. Unfortunately, this case in and of itself has limitations since it applies to rare instances of multiple primary malignancies and has issues with generalization to broader populations. However, the concept of requiring biopsy results to guide management is still key. Overall, cancer can be an elusive disease, and the possibility that the patient could develop multiple primary malignancies should always be considered.
